# The role of excimer light in dermatology: a review^☆^

**DOI:** 10.1016/j.abd.2023.12.007

**Published:** 2024-08-05

**Authors:** Dan Hartmann Schatloff, Catalina Retamal Altbir, Fernando Valenzuela

**Affiliations:** a Faculty of Medicine, Finis Terrae University, Santiago, Chile; b Faculty of Medicine, University of Chile, Santiago, Chile; c Dermatology Department, Clinical Hospital of the University of Chile, Santiago, Chile

**Keywords:** Alopecia areata, Atopic dermatitis, Lasers, excimer, Phototherapy, Psoriasis, Scleroderma, Vitiligo

## Abstract

Excimer light is a subtype of NB-UVB that emits a 308 nm wavelength, and can provide targeted phototherapy treatment. The absorption of 308 nm light by skin cells leads to therapeutic response in various common and ultraviolet-responsive skin diseases, such as psoriasis and vitiligo, and photo-resistant skin diseases such as prurigo nodularis, localized scleroderma, genital lichen sclerosis, and granuloma annulare, cutaneous T-cell lymphomas, among others. Excimer light has few adverse reactions and overall is well tolerated by patients, furthermore, it can be performed in places that are difficult to access. This article aims to explain the therapeutic bases and applications of excimer light in current dermatology.

## Introduction

The 308 nm (nanometer) excimer light is a widely used device throughout the field of dermatology in many diseases. The excimer laser emits a 308 nm wavelength that can provide targeted Ultraviolet B (UVB) treatment, and it can be used in difficult-to-reach sites, allows for site-specific dosing, and requires a lower cumulative UVB dose to achieve treatment efficacy.^1^ It is defined as the use of Ultraviolet (UV) radiation or visible light for the treatment of different diseases.^2^ The 308 nm excimer light is a type of phototherapy and a widely used device throughout the field of dermatology for many diseases.^3^

The excimer light uses a noble gas (Xenon) that decomposes in the presence of another reactive gas (Chloride), emitting UV radiation at a wavelength of 308 nm.^4^ When electrically excited, the gas mixture emits a monochromatic wavelength laser beam (308 nm), making exact, minute changes to irradiated material.^1^

There are two types of presentations of the excimer light: 1) Excimer laser, where laser phototherapy is directed and applied to the lesion through a tip with a spot of 14 to 30 nm in diameter, sparing healthy skin; 2) And excimer lamp that emits inconsistent light and, requires a longer time than a laser to emit the same fluency, this allows treatment in more extensive areas, with lower costs and easier transportation. The excimer lamp is smaller than the laser, it requires less space and has the advantage of portability. Both excimer laser and excimer lamps are more expensive than conventional NB-UVB phototherapy, and excimer laser is more expensive than excimer lamps and has higher operational and maintenance costs.^5^

This light is absorbed by keratinocytes, T-cells, and chromophores such as DNA, promoting DNA damage and reducing local inflammation and keratinocyte proliferation.^1^ This leads to the upregulation of the p53 tumor suppressor pathway^6^ and downregulation of the Bcl-2 proto-oncogene, leading to cell arrest and apoptosis.^7^

This mechanism translates into a combined action inhibiting dermal proliferation and an anti-inflammatory effect due to lymphocyte apoptosis. It also has an immunomodulatory activity which stands for the inhibition of antigen-presenting cells, the modification of the Th1 phenotype towards a Th2, and the induction of regulatory T-lymphocytes.^8^

It is theorized to promote repigmentation through melanocyte migration, activation, and production of melanin^1^ and is thought to occur due to elevated levels of peptide endothelin.^1^, ^4^

The following review seeks to summarize the main indications of the use of excimer phototherapy.

## Adverse effects and safety considerations

When using excimer phototherapy few adverse reactions have been reported and are consistent with other forms of phototherapy including phototoxicity with prolonged erythema, blistering, pruritus, tenderness and residual hyperpigmentation, and hypopigmentation.^3^

Referring to phototherapy in general, the most common effect is erythema, which occurs in up to 50% of therapeutic cycles but generates definitive suspension in only 2% of cases.^8^ An example of the distribution of these side effects is seen in a study performed on palmoplantar psoriasis, in which 20 of 54 patients presented prolonged erythema (48–72 hours) with a mild pruritic sensation, while only one patient presented vesicles and edema that resolved after local treatment with hydrocortisone 1% for three days and did not prevent the patient from completing therapy.^9^

To date, it has not been established a relationship between the doses received and the incidence of non-melanoma skin cancer,^10^ but it cannot be ruled out that prolonged follow-up of the patient's conditions an increase in carcinogenesis in the future.^11^

Also, there aren't studies that have associated photoaging with phototherapy, but this could be evaluated in clinical practice in patients who have received it for a long time.^12^ In any case, it is worth considering that the excimer light requires on average a smaller number of treatment sessions than conventional phototherapy and thus a smaller amount of cumulative UVB exposure, potentially reducing the patient’s cancer risk.^4^ In general excimer phototherapy is well tolerated by patients with fewer to no side effects.^1^

## Indications

The use of excimer light has been demonstrated in multiple conditions, the therapeutic effect of excimer light in the pathophysiological mechanisms related to different skin diseases is summarized in Table 1.

**Table 1:** Different skin conditions and the therapeutics effect of the excimer light in the pathophysiological mechanisms related.

Skin Pathology	Therapeutic effect
Psoriasis	Effective in difficult to treat psoriasis subtypes (palmoplantar pustular psoriasis, scalp, and nail psoriasis). In combination with topical corticosteroids.
	Treatment option in refractory to systemic treatment psoriasis.
	Can achieve full remission in >50% of patients.
Vitiligo	Useful in depigmentation of <10% total body surface area.
	Induces differentiation of melanocyte stem cells, stimulates production of melanin and proliferation and migration of melanocytes.
	In combination with topical immunosuppressants 75% of regimentation is seen.
Alopecia Areata	Achieves cosmetically acceptable hair regrowth (>50% of regrowth).
	Useful in cases of resistant Alopecia Areata.
	Decrease SALT score.
Mycosis Fungoides	Useful in early stages (lA, llA).
	Complete clinical response in 76.3% of cases.
Atopic Dermatitis	In atopic dermatitis involving <20% total body surface area.
	Decrease intensity of erythema, pruritus, excoriation and lichenification.
	Increases quality of life.
Granuloma Annulare	Achieves complete remission has been seen in combination with topical/systemic corticosteroids.
Nodular prurigo	Achieves complete remission in resistant nodular prurigo.
Morfea	Is useful to Inactivate the lesions and decrease lesion size.

### Vitiligo

Vitiligo is an acquired pigmentation disorder, characterized by the loss of epidermal melanocytes.^13^ Lesions on the face, neck, trunk, and proximal extremities are more sensitive to phototherapy, while those on the hands, feet, elbows, and knees are more resistant and with worse outcomes. A minimum of 6 months of treatment is required to assess the patient’s response to therapy.^5^, ^14^

NB-UVB phototherapy is the first-line treatment for the generalized form, and for localized disease, the excimer laser/lamp is more adequate.^14^ A 2016 meta-analysis compared the excimer laser, lamp, and NB-UVB without finding a significant difference in efficacy between these treatment modalities.^5^

The excimer light is indicated in patients with localized vitiligo less than 10% of Body Surface Area (BSA), localized non-segmental and segmental vitiligo, and has the advantage of not affecting the surrounding skin.^15^ Because of the smaller spot size, it is often preferred by patients and is reported to reduce the adverse event of hypopigmentation of healthy skin.^1^ Treatment sessions usually involve 2–3 sessions weekly,^16^ with a minimum of 48 hours between treatments, for a treatment duration of 4–36 weeks.^17^ This frequency of treatment (2–3 per week) appears to trigger earlier repigmentation of vitiligo patches and appears to depend ultimately on the total number of laser treatment sessions.^18^ It was discovered that the total number of treatments is the more important predictor of response.^1^

A meta-analysis comprising seven randomized controlled studies with 390 vitiligo patients showed no significant differences between 308 nm excimer laser and 308 nm excimer lamp when evaluating either >75% or >50% repigmentation rate, or between 308 nm excimer laser and NB-UVB on either 100% or >75% repigmentation rate. And more achieved >50% repigmentation when treated with 308 nm excimer laser than with NB-UVB (level of evidence 1+).^19^

The excimer light induces the differentiation of melanocyte stem cells, stimulates the production of melanin, and the proliferation and migration of melanocytes inducing repigmentation.^20^ It is recommended in combination with immunosuppressive treatment since it has shown a better improvement for >75% repigmentation when combined with topical tacrolimus.^21^ On the other hand, there is still insufficient evidence about the optimal regimen of therapy. A randomized study showed that a cyclic schedule (two months, twice weekly, one-month intervals) is equally effective to a continuous schedule of laser twice per week.^22^ In a pilot study of excimer light in 37 patients with vitiligo, the patients obtained initial repigmentation in the first 8 treatments and excellent repigmentation in 50% of patients at 6 months.^23^

Le Duff et al. reported equivalence between the excimer lamp and excimer laser in >50% repigmentation of vitiligo (p = 0.006). Lesion on the extremities and bony prominence showed the poorest response. Their treatment success rate was lower than the reported in other studies, results they attributed to the limited number of treatment sessions and the presence of lesion difficult-to-treat areas. They observed that excimer lamps are less expensive than lasers, allowing a more favorable cost/effectiveness ratio, although treatment takes a bit longer.^24^ Shi et al. conducted a randomized self-control trial with 14 patients with symmetrical vitiligo lesions, where one lesion was treated with 308 nm excimer laser and the counterpart with the 308 nm excimer lamp, these lesions were treated 3 times a week with the same dose both sides for a total of 20 sessions. The repigmentation rate was 50% achieved for 79% of the patches treated by laser and 87.5% of the patches treated by the lamp. The authors concluded that the two treatments exhibit similar results in terms of repigmentation with similar efficacies in treating vitiligo.^25^

The combination of excimer light with topical immunomodulators and fluticasone could enhance the clinical response to vitiligo, especially in resistant anatomical sites and in segmental vitiligo (level of evidence 1+).^16^ Topical calcineurin inhibitors, such as tacrolimus and pimecrolimus, khellin 4% ointment, hydrocortisone 17-butyrate cream, and tetrahydrocurcuminoid cream, have demonstrated improved efficacy when used in combination with excimer laser therapy.^1^

### Psoriasis

Psoriasis is a chronic, inflammatory disease that usually manifests with erythematous desquamative plaques. The excimer laser is currently indicated for adult and pediatric patients with mild, moderate, or severe psoriasis with <10% BSA involvement and the American Academy of Dermatology states the level of evidence is IIB.^3^

NB-UVB is considered the first-choice treatment for pregnant women with extensive disease.^13^ The NB-UVB induces apoptosis of pathogenic T lymphocyte and keratinocytes, leading to reduced epidermal regulatory hyperproliferation and local and systemic immunosuppression, and inhibits the Th17 pathway, increasing stability and restore regulatory T-cell function, and accumulated doses of this modality are believed to reduce levels of plasmin contributing to its therapeutic effects.^13^

The excimer laser/lamp is useful in the treatment of lesions affecting less than 10% of BSA, such as palms, soles, elbows, and knees.^26^ The excimer light is effective in difficult-to-treat psoriasis subtypes such as palmoplantar pustular psoriasis, scalp, nail psoriasis,^3^ and genitals.^1^ It is an effective treatment option in combination therapy with topical corticosteroids as well as a treatment option for psoriasis refractory to systemic treatment.^3^

It can be used to treat mild to moderate chronic plaque psoriasis in children.^1^

Usually, treatment is started with a dose of light 1‒3 times the minimal erythema dose with progressive increases in dose depending on the clinical results and tolerance.^16^ Excimer laser treatments typically occur 2–3 times a week, with a minimum of 48 hours between treatments, for 3–6 weeks. Clearance typically occurs within 8–10 treatment sessions, and maintenance therapy can be considered in patients with extensive disease.^1^

Bonis et al. were the first to report the efficacy of the excimer laser in 10 patients with chronic plaque psoriasis in a study comparing NB-UVB therapy and the excimer laser. This study reported that NB-UVB required an average of 30.1 treatment sessions to achieve clearance, compared with an average of 8.33 treatment sessions in patients treated with the excimer laser.^27^ A study performed in Rome included 54 patients with palmoplantar psoriasis considered 10 sessions (1 per week) and showed improvement after four sessions and complete remission in 57% after four months (24% partial remission and 19% moderate improvement) and these results were maintained at a 16 week follow-up.^9^

The excimer light can be used as monotherapy or in combination with other treatments, such as clobetasol propionate spray, flumetasone ointment, calcipotriol ointment, dithranol ointment, tacrolimus ointment, and 8-methoxypsoralen, increasing their efficacy.^1^ It has the same effectiveness as PUVA for the treatment of non-pustular palmoplantar psoriasis.^3^, ^28^

The use of excimer light is most appropriate in patients with psoriasis with a BSA of less than 10% and for plaques located in sites that are difficult to access with conventional phototherapies, such as the scalp and genitals.^1^

### Alopecia areata

The excimer light has been reported to be effective for alopecia areata. In most of these studies, patients had previously failed standard treatment, indicating the excimer laser´s utility in recalcitrant disease.^1^ A systematic review concluded that 50.2% of patients achieved cosmetically acceptable hair regrowth after an average of 12 weeks of treatment duration.^29^ Even in the case of resistant alopecia areata, the use of an excimer laser twice weekly for 12 weeks decreased the Severity of the Alopecia Tool (SALT) score, with a 100% response rate and a successful >50% regrowth of hair in 55.5% of patients.^30^ The studies mentioned above consider the use of excimer phototherapy as monotherapy.

It proposes that the induction of apoptosis of T lymphocytes results in reduced perifollicular inflammation and damage to the hair follicle.^31^ There is one study that evaluated the efficiency of phototherapy combined with topical minoxidil but compared the results to the use of minoxidil alone, showing that the combined treatment reduced the SALT score, and decreased the recurrence rate.^32^ Further studies comparing the results with the use of laser therapy alone should be performed to establish recommendations.

Alopecia areata can be treated with the 308 nm excimer laser, however, the laser has not shown improvement in alopecia universalis, alopecia totalis, or patches on the extremities.^4^

### Cutaneous T-cell lymphoma

The cutaneous T-Cell lymphomas (CTCL) are a heterogeneous group of non-Hodgkin’s lymphomas of the skin, with the mycosis fungoides (MF) subtype the most common variant.^13^ Multiple studies have shown that excimer laser is safe and effective; however, the follow-up was short, usually being reserved for sites not easily accessible to phototherapy or topical treatments such as acral surfaces or intertriginous areas.^33^

The latest guidelines of MF agree that in early stages,^34^ skin-directed therapies such as topical corticosteroids, UVB and PUVA localized radiation, and mechlorethamine represent the first therapeutic option.^34^, ^35^

The use of 308 nm excimer light in the treatment of stage IA to IIA MF has shown clinical and pathological benefits for patients with isolated lesions or lesions in areas that may be difficult to treat because of anatomic location.^36^ Referring to the effectiveness of this therapy It is estimated a 73.6% complete clinical response rate to excimer therapy in cases of folliculo-tropic and palmaris et plantaris mycosis fungoides.^37^ In a study by Nitisco et al., 5 patients treated with the excimer laser achieved complete remission with less than 10 treatment sessions.^38^

A prospective controlled study evaluated the efficacy of the 308 nm excimer laser in 8 patients with stage IA or 1B of MF, who were refractory to topical steroids, the patients received 20 treatments with laser over 10 weeks and follow-up for a minimum of 30 months. At the end of the study, 3 patients had a complete clinical and histological response and remained free of disease after 30 months, 3 patients had a complete clinical and histological clearance of disease but with relapse within 30 months, and one patient withdrew and the other had a partial response.^39^

In 2019 Armenta reported an interesting case in which excimer light was preferred rather than generalized phototherapy in a patient with stage IA MF with a history of dysplastic nevi and proposed target therapy as a safer alternative in patients with conditions that put them in a higher risk of developing skin cancer.^40^

### Atopic dermatitis

Atopic dermatitis (AD) is an inflammatory, recurrent, and relapsing chronic disease, characterized by the presence of eczema and pruritus. Phototherapy is useful in the treatment of moderate to severe AD. The currently used modalities are NB-UVB, UVA1, PUVA, and excimer laser/lamp.^41^ FDA approved for the treatment in both adults and children.^1^

In 2006 Baltás published a case report of 15 patients with atopic dermatitis affecting less than 20% of BSA. Patients were treated with excimer light twice weekly and after 1 month of phototherapy, the clinical scores considering the intensity of erythema, excoriation, and lichenification as well as the quality of life and pruritus were significantly lower than the initial values.^42^

As to the mechanism by which this occurs, it was later described in a study carried out by Kurosaki that the excimer light significantly changed the composition of the bacterial microbiome in the lesional skin of atopic dermatitis, decreasing the colonization of *Staphylococcus aureus* in the skin.^43^ These improve the skin barrier function, reducing pruritus and tissue inflammation. Immunomodulatory effects include decreased expression of IL-5, IL-13, and IL-31, as well as the induction of T-cell apoptosis and dendritic cell reduction.^13^, ^41^

Excimer lasers used for 10 weeks have shown good results compared to clobetasol propionate. The excimer lamp in combination with emollients resulted in AD severity score improvement within 4 weeks.^41^

A study investigating the prurigo form of AD compared the excimer laser with clobetasol propionate 0.05% ointment. Both treatments demonstrated efficacy, but excimer lasers showed more improvement at follow-up. This study concluded that the excimer laser is an effective alternative to topical corticosteroids and can be used in patients with recalcitrant disease.^44^ Another study evaluated the efficacy of excimer laser in adults and children with AD and reported that 66.7% of patients achieved complete remission with 6–12 treatments, and 44% of patients maintained results at 16 weeks.^45^

### Scleroderma

Morphea is a localized form of scleroderma, a chronic autoimmune cutaneous connective tissue disease, it is characterized by the presence of inflammation, excessive production of collagen, and limited sclerosis of the skin.^13^, ^46^

In the treatment of localized scleroderma or morphea, excimer light has been shown to inactivate lesions and decrease their size alone and in combination with systemic (methotrexate) or topical therapy (calcipotriene/betamethasone). The excimer laser expresses multiple advantages such as a limited course of treatment, lower UV dose exposure, and a direct way of acting.^46^, ^47^

The efficacy of excimer laser in morphea mainly results from their anti-inflammatory action, due to the action of the 308 nm wavelength inducing DNA breakage in lymphocytes.^48^ The keratinocytes and T-cells absorb the light, promoting DNA damage and reducing local inflammation and keratinocyte activity.^1^

Hanson et al. reported a case of linear scleroderma in an adolescent woman treated with methotrexate and excimer laser, with a decrease in the lesion size and relief of symptomatic discomfort by 7 months.^49^ Nitisco et al., observed that 3 of 5 patients with morphea with the use of excimer light experienced partial clinical remission with marked improvement in skin texture after 8–12 treatment sessions, however, it was accompanied by residual hyperpigmentation.^47^

Tatu et al., in their review, recommended 308 nm excimer laser therapy as an effective and safe treatment of superficial inflammatory morphea lesions (i.e., erythematous plaque or oedematous patches), especially when the topical corticoids or UVA therapy do not give clinical improvement. If the therapy with UVB or NB-UVB is recommended, the excimer laser represents a valuable therapeutic alternative.^48^ In another systematic review, the authors analyze the different laser therapies in the treatment of morphea and conclude that excimer laser can be considered in active short-lasting inflammatory lesions (erythematous plaque) and active short-lasting but sclerotic lesions.^46^

Phototherapy is safe, as its effect is directed at the skin, without the risk of systemic complications, and its effectiveness depends on the applied UVR doses. The excimer phototherapy it’s an alternative to those with contraindications to immunosuppressive therapy and benefits from the method.^13^

### Other conditions

Prurigo nodularis is a skin disease that is known to be resistant to a variety of treatments. Several reports show that excimer phototherapy is effective for this condition in patients with no response to oral or intralesional corticosteroids. In one of these studies, complete remission was achieved with an average of 10 sessions and there was a significant decrease in sleep disturbance and pruritus.^50^, ^51^ In 2022. Mitsubishi reported a case of chronic nodular prurigo that improved with combined therapy with excimer light and systemic corticosteroids.^52^

A case report of a patient of 17 years with a recalcitrant form of perioral dermatitis. She had been treated with several topical agents including topical steroids and calcineurin inhibitors with no improvement. The patient was treated with 2 sessions and no recurrences in the following 3 months.^53^

In 2012, Bronfenbrener presented a case of a 73-year-old woman with a long history of granuloma annulare (GA) with previous unsuccessful therapy with response to PUVA/methotrexate, isotretinoin and topical and intralesional steroids injections, it was treated with excimer laser therapy responding with complete resolution after 15 sessions with no residual scarring.^54^ In another report of a patient with a 40-year history of GA, complete remission was achieved with 15 treatments with the excimer laser.^55^ Later, in 2023 Mizawa reported two cases of generalized GA that were successfully treated with 8 (one per week) and 9 (one per month) sessions of excimer phototherapy associated with topical and systemic corticosteroids.^56^ In a recent systemic review comparing various light- and laser-based entities for GA, the authors recommended that UVB/nbUVB/excimer laser therapy should be considered as a first-line treatment for patients with generalized GA for its effectiveness and favorable long-term safety profile in comparison with PUVA and photodynamic therapy (PDT).^57^

The excimer light has also been used to treat scars and striae with good results, helping repigmentation of hypopigmented scars. With the advantages of being widely availed, improved repigmentation with minimal invasion. In many cases, the management can start 2 weeks after the initial wound formation, but in most cases, it starts 3 months after surgical revision.^58^, ^59^

The excimer laser has demonstrated efficacy in hypopigmented conditions including nevus depigmentosus, idiopathic guttate hypomelanosis, lichen striatus, and pityriasis alba.^60^ A case report of an infant with facial nevus depigmentosus reported repigmentation of the lesion after 10 treatments.^61^

## Discussion

Excimer light is a subtype of NB-UVB that emits a 308 nm wavelength that can provide targeted phototherapy treatment.^1^, ^13^ The Food and Drugs Administration (FDA) approved the excimer light for the treatment of psoriasis, vitiligo, atopic dermatitis, and leukoderma, and has demonstrated efficacy in the treatment of other hypopigmented disorders, cutaneous sclerosis, allergic rhinitis, alopecia areata, leukoderma, prurigo nodularis, cutaneous T-cell lymphoma, CD30+ lymphoproliferative disorders, oral lichen planus with erosive disease, granuloma annulare, localized scleroderma, as an adjuvant therapy in adult cutaneous Langerhans cell histiocytosis.^1^, ^4^, ^13^, ^16^

Target phototherapy had good responses seen in localized involvement, resistant lesions, and in children in whom their use is more accepted and convenient (evidence IIB).^43^ It can apply directly to the lesion, sparing healthy skin; this allows higher doses to be administered from the beginning, in localized usually less than 10% of BSA and chronic inflammatory dermatosis.^13^, ^43^, ^62^ It offers the advantage of limiting UVB exposure to the affected skin, can be used in difficult-to-reach sites, allows for site-specific dosing, and requires a low cumulative UVB dose to achieve treatment efficacy,^1^ such as the scalp and palmoplantar skin.^13^

Is a well-tolerated treatment option for patients, with few adverse reactions that have been reported localized to the area treated, including erythema, blistering, tenderness, pruritus, hyperpigmentation, and hypopigmentation. Fewer adjuvant treatments are needed, and long-term side effects are reduced.^1^, ^3^ Excimer light requires a reduced number of treatment sessions and lower cumulative UVB exposure, effectively minimizing potential side effects.^3^ Importantly, there is no published evidence suggesting an increased risk of skin cancer associated with therapeutic excimer light use.^1^

Its versatility extends to the treatment of various dermatological conditions, including psoriasis, vitiligo, atopic dermatitis, alopecia areata, and more. Furthermore, its applicability to challenging anatomical sites, such as the scalp, palms, and soles, underscores its adaptability. With region-specific dosing capabilities, it caters to the individualized needs of patients, particularly those with recalcitrant lesions.^1^

In a recent study in vitro, Hu et al demonstrated the protective potential of 42 °C heat stress pretreatment on human melanocytes subjected to 308 nm laser-induced DNA damage in vitro. This study revealed that melanocytes, exposed to a one-hour heat treatment at 42 °C, displayed no cytotoxic effects, attenuating the adverse impacts caused by the 308 nm excimer laser, as indicated by a reduction in DNA damage and the amelioration of cell apoptosis. The observed protective outcomes were closely linked to both the level and subcellular localization of Heat Shock Protein 70 (HSP70). The implications suggest that the integration of preheat treatment with the 308 nm laser could serve as a promising therapeutic avenue, not only for enhancing repigmentation in vitiligo but also for mitigating the potential for UVB-induced photo-damage. In conclusion, these findings contribute valuable insights to the exploration of innovative approaches aimed at improving the safety and efficacy of vitiligo phototherapy.^63^

Although not a primary treatment option, the excimer light remains an excellent choice for patients with limited skin disease, offering a well-established and efficacious approach.^3^ The excimer light holds significant promise in enhancing the therapeutic landscape of dermatology.

In the two types of presentation of the excimer light, lamp, and laser, both have shown similar or superior effectiveness to NB-UVB in the treatment of psoriasis and vitiligo,^13^ treating small areas, and avoiding unnecessary exposure of normal skin to radiation.^5^ Long-term effects of UV phototherapy include photoaging and photodamage, which are reduced with the excimer laser due to the reduced cumulative dose required.^1^

It’s important to understand that before indicating phototherapy, a complete patient assessment should be performed searching for possible contraindications and reaffirming that the patient can come to the treatment at least twice a week because one of the main method limitations is the difficulty that patients must attend the sessions.^13^ The excimer laser is an increasingly popular treatment modality and an important dermatologist tool. The role of the excimer laser in dermatology will continue to expand as more studies investigate its application in different diseases.

## Conclusion

In conclusion, the excimer light stands as a highly promising therapeutic approach within the field of dermatology. It has demonstrated efficacy in many dermatological diseases, with specific indications and few adverse reactions. The role of the excimer light in dermatology will continue to expand as more studies investigate its application in different diseases. However further studies are needed to evaluate the long-term safety of the excimer light.

## Financial support

None declared.

## Authors’ contributions

Dan Hartmann Schatloff: Approval of the final version of the manuscript; critical literature review; manuscript critical review; preparation and writing of the manuscript.

Catalina Retamal Altbir: Critical literature review; manuscript critical review; preparation and writing of the manuscript.

Fernando Valenzuela: Approval of the final version of the manuscript; critical literature review; effective participation in research orientation; manuscript critical review; preparation and writing of the manuscript.

## Conflicts of interest

None declared.
